# Exome sequencing revealed DNA variants in *NCOR1*, *IGF2BP1*, *SGLT2* and *NEK11* as potential novel causes of ketotic hypoglycemia in children

**DOI:** 10.1038/s41598-020-58845-3

**Published:** 2020-02-07

**Authors:** Yazeid Alhaidan, Martin J. Larsen, Anders Jørgen Schou, Maria H. Stenlid, Mohammed A. Al Balwi, Henrik Thybo Christesen, Klaus Brusgaard

**Affiliations:** 1 Department of Clinical Genetics, Odense University Hospital, 5000 Odense C, Denmark; 2 Department of Clinical Research, Faculty of Health Sciences, University of Southern Denmark, 5000 Odense C, Denmark; 3 Department of Medical Genomics Research, King Abdullah International Medical Research Center, Riyadh, 11426 Saudi Arabia; 4 King Saud bin Abdulaziz University for Health Sciences, Riyadh, Saudi Arabia; 5 Hans Christian Andersen Children’s Hospital, Odense University Hospital, 5000 Odense C, Denmark; 6 Department of Paediatric Endocrinology, Uppsala University Children’s Hospital, Uppsala, Sweden; 7 Odense Pancreases Center, www.OPAC.nu, Uppsala, Sweden; 8 Near East University, Nicosia, Cyprus

**Keywords:** Diagnostic markers, Metabolic syndrome, Metabolic disorders, Genetics research, Paediatric research

## Abstract

Unexplained or idiopathic ketotic hypoglycemia (KH) is the most common type of hypoglycemia in children. The diagnosis is based on the exclusion of routine hormonal and metabolic causes of hypoglycemia. We aimed to identify novel genes that cause KH, as this may lead to a more targeted treatment. Deep phenotyping of ten preschool age at onset KH patients (boys, n = 5; girls, n = 5) was performed followed by trio exome sequencing and comprehensive bioinformatics analysis. Data analysis revealed four novel candidate genes: (1) *NCOR1* in a patient with KH, iron deficiency and loose stools; (2) *IGF2BP1* in a proband with KH, short stature and delayed bone age; (3) *SLC5A2* in a proband with KH, intermittent glucosuria and extremely elevated p-GLP-1; and (4) *NEK11* in a proband with ketotic hypoglycemia and liver affliction. These genes are associated with different metabolic processes, such as gluconeogenesis, translational regulation, and glucose transport. In conclusion, WES identified DNA variants in four different genes as potential novel causes of IKH, suggesting that IKH is a heterogeneous disorder that can be split into several novel diseases: NCOR1-KH, IGF2BP1-KH, SGLT2-KH or familial renal glucosuria KH, and NEK11-KH. Precision medicine treatment based on exome sequencing may lead to advances in the management of IKH.

## Introduction

Hypoglycemia is the most common metabolic disease beyond infancy. Despite its frequency, the definition of hypoglycemia has been discussed for decades, and hypoglycemia “remains one of the most confused and contentious issues”, as stated by Cornblath *et al*.^1^. In a more recent consensus guideline, the lower limit of average plasma glucose values beyond three days of age was given as 3.9 mmol/L as in adults, although no single cut-off level for p-glucose to cause brain injury can be provided for several reasons, including the variable presence of ketone bodies as an alternative fuel^2^.

Hyperketotic hypoglycemia (KH) can occur in nondiabetic infants and children as a result of growth hormone deficiency, adrenal insufficiency, or disorders of glucose metabolism with intact fatty acid oxidation, including glycogen storage disease (GSD) type 0 [OMIM: 240600], III [OMIM: 232400], VI [OMIM: 232700] and IX [OMIM: 306000-261750-613027]^3–9^. Excluding such diagnoses, ketotic hypoglycemia after prolonged fasting in younger children has been categorized as idiopathic ketotic hypoglycemia (IKH) or accelerated starvation^10–12^. IKH debuts from approximately 7 months of age and usually resolves around 6–7 years^11–14^. The overall prevalence of non-diabetes-related hypoglycemia is incompletely understood. Several hypotheses have been raised. One of which may argue that this might be a physiological phenomenon, representing the lower tail of the Gaussian distribution of fasting tolerance^13^. Although emergency departments showed an incidence of 3.9–4.0/100,000 admissions due to IKH^11,15^, the estimated prevalence of the different genetic disorders leading to hypoglycemia may vary considerably between populations and according to subtypes.

Here, we investigate nine families with proposed IKH, applying a multistep screening strategy based on trio exome sequencing, to potentially identify a cause of IKH and hence improve treatment within the conceptual frame of personalized medicine.

## Results and Discussion

### Exome analysis

Out of 38 patients with hypoglycemia, 26 cases were excluded as follows: 5 patients (13%) were diagnosed with a known GSD variant, 5 patients (13%) were diagnosed with hypoketotic hypoglycemia, and 16 cases (42%) were excluded due to the lack of informed consent. In the remaining ten patients (boys, n = 5; girls, n = 5) from nine families, the adult family members were either healthy, affected or had a history of hypoglycemia attacks. The phenotype and symptoms, including laboratory results, are presented in Table 1. Trio analysis resulted in a list of four single base variants distributed between different genes in four families (Table 2).

**Table 1:** Patient clinical information in 10 children with IKH.

Family ID	Sex	Onset hypoglycemia	Lowest recorded blood glucose (mmol/L)	Highest ketone bodies (mmol/L or urine stick)	Other abnormal lab results	Familial hypoglycemia	Age at last follow-up	Treatment at last follow-up
HH16	F	22 m	1.8	4.9	Chronic iron deficiency and loose stools	None	5 y, 4 m	Dietary, cornstarch Continuous maltose by gastrostomy Sandostatin LAR Sirolimus
HH21	M	12 m	1.5	4+ Urine dipstick	Short stature (−2.54 SD), bone age 3.7 years delayed	None	11 y, 6 m	Dietary, cornstarch
HH24	M	2 y, 2 m	2.1	3.1	IGF-I and IGF-BP3 low in the normal range Short stature (−2.1 SD), normal bone age	Father hypoglycemia-like episodes in childhood	5 y, 11 m	Dietary, cornstarch
HH26	M	18 m	1.1	1.8	↑ GLP-1 4 + glycosuria upon 15 h of fasting	None	7 y, 8 m	Clinical remission
HH28	F	4 y, 10 m (first documented, suspicion from 1½ y)	1.5	2.6	Blood glucose was up to 31.6 mmol/L while nonketotic Born preterm (33 weeks) and weighed 2000 g	Brother and mother blood glucose 1.4 (1.7) −28 (32.4) mmol/L, hypoketotic Normal HbA1c. Maternal grandfather had hypoglycemia-suspected attacks	7 y, 3 m	Dietary (avoidance of sugar-rich food items)
HH30	M	19 m	1.2	2.9	None	Mother ketotic hypoglycemia. Her two siblings and their mother suspected to have hypoglycemia as children	3 y, 7 m	Dietary, cornstarch, Sandostatin LAR
	F	18 m (first documented)	0.7	2.9	None		2 y, 2 m	Cornstarch, continuous maltose in gastrostomy during sleep
HH31	M	5 y (first documented)	2.3	2.3	↑APTT ↑INR ↑p-ammonia ↓IGF-1 ↓IGF-BP3 Height -1.5 SD A migraine, tremor and mild retardation	None	8 y, 9 m	Dietary, cornstarch GH treatment despite no GHD
HH32	F	13 m	2.2	4.7	None	None	3 y, 8 m	Dietary, corn starch
HH37	F	20 m	1.4	4.9	Short stature (−2.0 SD) bone age -2,3 y low-normal p-IGF-1	None	8 y, 4 m	Dietary, cornstarch GH treatment

Abbreviations: GHD; Growth Hormone Deficiency. FFA; Free Fatty Acid. SD; Standard Deviation. APTT; Activated Partial Thromboplastin Time. INR; International Normalized Ratio.

All patients had an exclusion of muscular and liver diseases (including intramuscular glucagon test), pituitary (gh) and adrenal insufficiency unless otherwise specified.

**Table 2:** Positive exome sequencing results in four families.

Family ID	Gene	RefSeq ID	Change			NFE - Freq.	HGMD	SIFT/PolyPhen-2/PROVEAN Prediction^a^
			Coding DNA	Amino Acid	Zygosity			
HH16	*NCOR1*	NM_006311.3	c.4564 A > G	p.(Thr1522Ala)	Paternal heterozygous	1.6 × 10^−5^	NR	D/D/D
HH21	*IGF2BP1*	NM_006546.3	c.1501 C > T	p.(Arg501Trp)	*de novo*, heterozygous	9 × 10^−6^	NR	D/D/D
HH26	*SLC5A2*	NM_003041.3	c.198 + 6 A > G	—	Maternal heterozygous	1.6 × 10^−5^	NR	—
HH31	*NEK11*	NM_001321221.1	c.1844A > G	p.(Glu615Gly)	Paternal heterozygous	NR	NR	D/D/D

Abbreviations: NFE-freq; Non-FinishEuropean Frequency by the Genome Aggregation Database (gnomAD). NR = Never Reported.

^a^D: Deleterious.

### The role of *NCOR1* in gluconeogenesis activity

A nonsyndromic, normal-weight Caucasian girl presented with p-glucose of 1.8 mmol/L and blood ketones 4.9 mmol/L at 22 months of age during an infection episode (HH16, Table 1). Repeat similar attacks were subsequently frequent. Routine hormonal and metabolic screening including glucose response to i.m. glucagon was normal. Upon further investigations, blood ketones could fall to 0.0 mmol/L during hypoglycemia attacks. Fasting was not tolerated for 1.5–2 hours on several occasions. In contrast, fasting could be tolerated for 10 hours after overnight gastric infusion of maltose. Upon fasting provocation with p-glucose 3.3 mmol/L, p-free fatty acids (FFAs; aliphatic carboxylate C6-C20) were elevated, 2.78 (ref. 0.1–0.75) mmol/L. Simultaneously, p-triglycerides were normal, 0.94 mmol/L, but p-insulin was not suppressed, 66 (ref. 18–173) pmol/L. The lowest p-insulin recorded without medication was 33 pmol/L at p-glucose 4.9 mmol/L. During ketotic hypoglycemia attacks, the patient was treated with i.v. glucose, which led to a pronounced increase in the glucose demand to 11 mg/kg/min to maintain normoglycemia, suggesting rapid glucose elimination. Oral glucose tolerance test (OGTT) and a trial with fruit juice 250 ml, containing 23 g of carbohydrate led to prompt illness with p-glucose 3.8–3.9 mmol/L, ketosis, but normal p-lactate and pyruvate. Morning fasting p-glucagon was increased, 59 (ref. 5–20) pmol/L during normoglycemia. Liver counts and hepatic ultrasound were normal, and neutrophilic counts and enterocyte autoantibodies were only transiently affected. Liver biopsy showed massive glycogen storage in hepatocytes with decreased numbers of mitochondria and lysosomes. Gastroscopy and coloscopy were macroscopically normal, but colon biopsies showed moderate inflammation without crypt abscesses or granulomas. Normal investigations included muscle biopsy with analysis of respiratory chain enzymes, mtDNA in blood, muscle and fibroblasts, isoelectric transferrin pattern and carbohydrate-deficient transferrin.

Her chronic iron deficiency and loose stools with a positive reaction for blood had onset before treatment for hypoglycemia. Screenings for celiac disease and lactose intolerance were negative.

Dietary treatment including continuous maltose gastric infusion was, however, unsuccessful in avoiding often severe symptoms from hyperketosis and hypoglycemia with numerous hospitalizations, which is why off-label medical treatment was tried with diazoxide, long-acting octreotide, sirolimus, and continuous s.c. or i.v. glucagon up to 50 mcg/kg/h, without or with partial effect. p-Glucagon was >100 pmol/L in five measurements 14–16 hours after discontinuation of s.c. glucagon on two other occasions, suggesting a very prolonged half-life of glucagon.

Both parents were found to have normal fasting plasma glucose, p-glucagon and HbA1c, no ketone bodies and no history of hypoglycemia symptoms. Of note, the father had cholesteatoma.

The exome scan revealed a novel, rare, paternal heterozygous variant p.Thr1522Ala in the transcriptional repressor gene Nuclear Receptor Corepressor 1 (*NCOR1*) (HH16, Table 2). By SIFT, PolyPhen-2, and PROVEAN prediction software analyses, the variant was located in the highly conserved region up to Tetraodon and predicted to be deleterious. In addition, no changes were observed in the copy number variant analysis, and no other candidate genes were identified. No human reports of *NCOR1* variants have been published.

**Figure 1:**
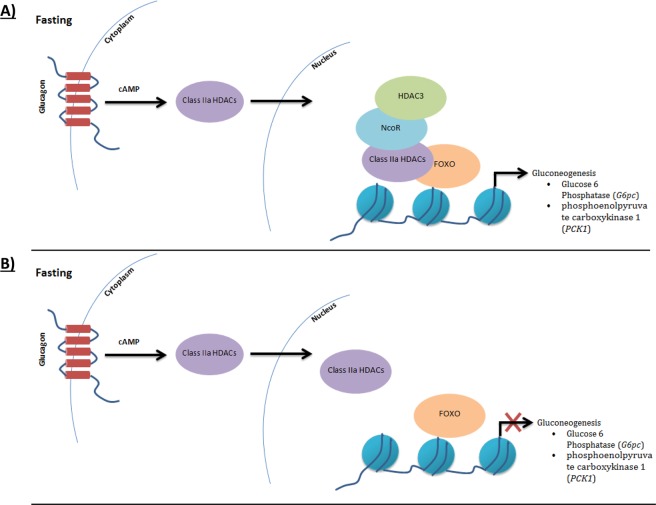
NCOR/HDAC3 recruitment for transcriptional induction of gluconeogenesis pathway. Molecular models of NCOR/HDAC3 recruitment for gluconeogenesis transcriptional genes during fasting. (**A**) In present of NCOR/HDAC3 complex, gluconeogenesis activated via FOXO/Class IIa HDACs. (**B**) in absent of NCOR/HDAC3 complex, Class IIa HDACs lose it is ability to activate FOXO leading to suppress gluconeogenesis transregional genes.

The NCOR1 complex activity has previously been shown to be dependent on the histone deacetylase-3 (HDAC3) interaction^16^. The (p.Thr1522Ala) variant in *NCOR1* is located in the domain interacting with *HDAC3*^17^. HDAC3 binds NCOR1 in three main domains that must be present together for an active form of class I HDACs, including the NCOR1 fragment containing amino acid residues 1469-1740^18^. The active form of class I HDACs was reported to be recruited by class IIa HDACs, which act as a complex with FOXO regulating the metabolic function of liver phosphoenolpyruvate carboxykinase 1 (*PCK1*) and glucose 6-phosphatase catalytic (*G6PC*) (Fig. 1)^16,19–21^. Interestingly, class IIa HDACs alone were unable to stimulate *in vitro* deacetylation of FOXO1 alone, causing glycogen accumulation with a similar characterization of GSD Ia, unlike recombinant NCOR1/HDAC3 complex^21^. In addition, C57BL/6 mutant mice with the variant p.Tyr478Ala in the deacetylase activation domain (DAD) of *NCOR1* produce a mutant protein that is stable but unable to associate with or activate Hdac3, leading to a reduction in hepatic glucose production and an increase in ketones and fatty acids^22^.

In addition, the NCOR1/HDAC3 complex was reported to regulate hepcidin expression. The liver-expressed peptide hormone hepcidin, encoded by *HAMP*, was found to be regulated by the NCOR1/HDAC3 complex by binding to its promoter^23^. In the Huh7 human hepatoma cell line, downregulation of either *NCOR1* or *HDAC3* individually increased *HAMP* expression^23^. Overexpression of hepcidin in mice causes iron deficiency anemia by inhibiting intestinal iron absorption and limiting the release of stored iron^24^.

Here, we present the first human report of a potentially pathogenic *NCOR1* variant. We hypothesize that the p.Thr1522Ala variant in *NCOR1*, located on the site where the interaction with HDAC3 occurs, affects the activity of the class I HDAC complex (NCOR/HDAC3), subsequently causing the loss of the class IIa HDAC complex activity, leading to suppression of glucose production pathways, increased glycogen storage, p-triglycerides, and p-glucagon and iron deficiency through overexpression of hepcidin. The carrier father was healthy except for cholesteatoma, suggesting a broad phenotypic spectrum with reduced penetrance or variations in the genetic background in keeping with the concept of synergistic heterozygosity^25^.

The clinical similarities with GSDIa [OMIM: 232200] and GSDIb [OMIM:232220] (KH, liver glycogen storage, iron deficiency, loose stools) and the effect of the NCOR/HDAC3 complex on G6PC suggest that NCOR-KH may represent a novel type of GSDIa, GSDId, despite the normal liver routine parameters and liver size at the present age.

The results of the exome sequencing analysis led to an increased focus on carbohydrate supply and treatment with a low-glycemic index diet and modified high amylopectin cornstarch (Glycosade)^26^.

### Role of *IGF2BP1* in hypoglycemia and the *IGF2* level

A nonsyndromic, nonobese Caucasian boy presented at the age of 12 months with severe ketotic hypoglycemia (HH21, Table 1). The parents were of normal height [mother 157.8 cm (−1.6 SD), father 175.3 cm (−0.8 SD)], had no history of hypoglycemia or delayed constitutional growth and puberty, and normal fasting p-glucose and HbA1c. The boy was born at term, with a birth weight of 3500 g and birth length of 53 cm.

Of special note, the boys’ height declined from −1.22 SD at four years to −2.54 SD at 11.6 years old, where he was prepubertal with a bilateral testicle volume of 3 mL and no pubic hair. By repeat X-ray, the bone age was increasingly delayed, with a delay of 2.2 to 4.2 years. Normal investigations included repeat p-IGF1 (−0.4 to 1.32 SDS), p-IGFBP3 (+0.15 to +0.31 SDS), GH-stimulation tests (peak GH by arginine 25 mU/L; clonidine 35 mU/L), ACTH stimulation test, p-insulin suppression during hypoglycemia, thyroid parameters, liver counts, hepatic ultrasound, i.m. glucagon test, and metabolic screenings. No abnormalities were found in array CGH.

**Figure 2:**
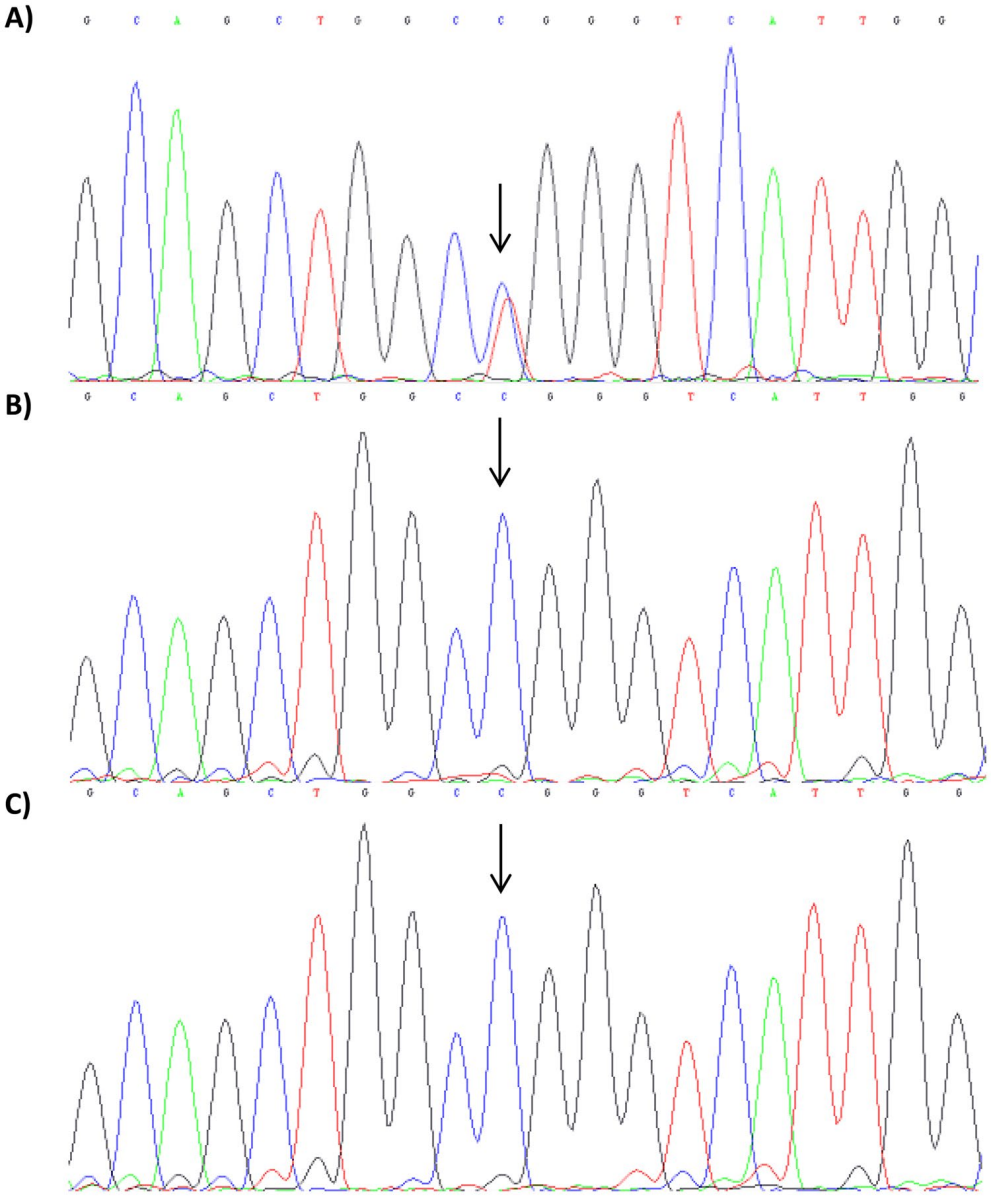
HH21 family’s Sanger sequencing results for IGF2BP1 gene. Sanger sequencing results; (**A**) proband shows a heterozygous de novo mutation in location c.1501 C > T; p.Arg501Trp. The corresponding; (**B**) paternal and (**C**) maternal sequence presented wild-type alleles of IGF2BP1.

**Figure 3:**
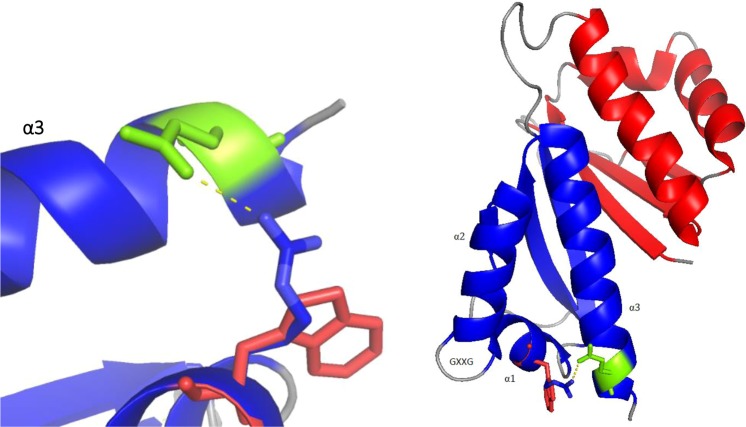
3D protein structure. The arginine (blue) in α1 is mutated into a Tryptophan (red). Due to this mutation, the hydrogen bonds (yellow dotted lines) between arginine and glutamine in α3 (green) are lost.

The exome analysis identified a *de novo* variant (p.Arg501Trp) in the insulin-like growth factor 2 mRNA-binding 1 (*IGF2BP1*) gene (HH21, Table 2). The p.Arg501Trp variant was validated using Sanger sequencing (Fig. 2). Arginine is a highly conserved amino acid up to *Caenorhabditis elegans*. Prediction tools such as SIFT, PolyPhen-2, and PROVEAN predicted this variant to be disease-causing. In addition, no changes were observed in the copy number variant analysis, and no other candidate genes were identified. 3D protein structure analysis showed that the positively charged arginine position 501 in α1 created a side chain hydrogen bond with glutamine position 559 in α3 (Fig. 3). Hence, the Arg501Trp variant weakens the protein structure by the loss of this bond when position 501 is substituted with nonpolar aromatic tryptophan. In addition, Chao *et al*., who deposited the protein structure (PDB-ID: 3KRM), showed that the model of the RNA binding site on the surface of KH4 was located on the mutant side^27^. No humans with germline *IGF2BP1* variants have been published.

*IGF2BP1* is involved in cell growth and differentiation during development and regulates *IGF2*, which encodes a protein containing four K homology domains (KH1-4) and two RNA recognition motifs. Of note, *IGF2BP1* should be discerned from *IGFBP-I* and other IGF binding protein genes. *IGF2BP1* is functionally involved in cell growth and differentiation during development and functions as a translational regulator of a number of proteins, including *IGF2*. *In vitro, IGF2BP1* suppresses the translation of *IGF2* mRNA by binding to its 3′-UTR^28,29^. Hence, loss of function will lead to increased IGF2 levels.

IGF2 is known to bind and activate insulin receptors (IRs)^30,31^. Several studies in tumor tissues find that continuous activation of IRs by IGF2 will eventually lead to suppression of free fatty acids and inhibition of glucose release, gluconeogenesis, and glycogenolysis in the liver causing hypoglycemia^32–34^. In hepatocellular carcinoma, patients with significant elevation of IGF2 had severe hypoglycemia and suppressed insulin secretion^35^. Additionally, *Igf2bp1*^*−/−*^ mice exhibit growth retardation from E17.5 and a postnatal growth deficiency of 40% compared to wild-type^36^. Our *IGF2BP1* variant patient had normal birth length but decreased postnatal height to −2.54 SD at 11 years, consistent with a milder heterozygous phenotype.

Taken together, we present the first report of a human *IGF2BP1* variant. We suggest that a heterozygous *IGF2BP1* variant affects the glucose production pathways leading to ketotic hypoglycemia and short stature. The exome sequencing results led to expanded investigations of the patient’s GH-IGF axis and a trial of off-label GH treatment.

### Familial renal glucosuria causing hypoglycemia

An 18-month-old nonsyndromic, nonobese boy presented with severe ketotic hypoglycemia with loss of consciousness and convulsion and blood glucose of 1.1 mmol/L at onset (HH26, Table 1). His parents were nonconsanguineous Swedish Caucasians and healthy with no history of hypoglycemia and normal fasting p-glucose and HbA1c. Upon follow-up investigations, an i.v. glucose tolerance test showed increased glucose elimination, a K-value of 7.7, and a p-glucose half-life of nine minutes. At 3.3 years of age, 15 hours of fasting led to KH with p-glucose of 2.3 mmol/L and blood ketones of 1.8 mmol/L. The somnolescent patient was rescued with i.v. glucose, (positive Whipple’s triad). The first following urine test showed +2 ketonuria and, surprisingly, +4 glucosuria by urine stix despite a maximally recorded p-glucose of 6.2 mmol/L.

Samples from the end of fasting at a p-glucose of 2.8 mmol/L showed suppressed p-insulin (<2 pmol/L) and an appropriate glucagon response with p-glucagon of 74 pmol/L. The p-lactate, p-triglyceride and cortisol response (764 nmol/L) were normal. His p-HbA1c was low, at 28 mmol/mol. By i.m. glucagon test, a high response of p-glucose from 5.2 to 11.6 mmol/L was observed with subsequent symptomatic hypoglycemia, namely, blood glucose of 3.1 mmol/L. By standard OGTT, p-glucose rose from 5.4 to 7.0 mmol/L. The peaks of p-insulin (226 pmol/L), p-C-peptide (2115 pmol/L), and p-proinsulin (21 pmol/L) were relatively blunted compared to those of the normal adult range. p-GIP showed a modest rise from 12 to 37 pmol/L, while p-glucagon fell from 12 to 7 pmol/L and p-GLP-1 declined from 118 pmol/L at fasting time 0 min (normal fasting value < 30 pmol/L), to over 73 pmol/L (30 min), and finally to 29 pmol/L (60 min).

Normal findings included growth, p-GH, IGF1 and IGFBP3, p-carnitine profile, and p- and u-metabolic screenings. Bilateral adrenal hemorrhage with calcifications was observed by CT scan after the initial severe hypoglycemic episode with convulsions. His later normal cortisol response to fasting and a normal ACTH stimulation test excluded adrenal insufficiency. Additionally, urine adrenalin and noradrenalin values were normal. Calcium-phosphate metabolism parameters were found to be in the normal range. On follow-up to age seven years and eight months, he was in clinical remission with normal blood glucose values.

The boy’s exome analysis showed a maternally inherited splicing variant (c.198 + 6 A > G) in the Solute Carrier Family 5 Member 2 gene (*SLC5A2*), which encodes the sodium glucose co-transporter 2 (SGLT2) and is known to cause familial renal glucosuria (FRG [OMIM: 233100]) (HH26, Table 2). NetGene2, MaxEnt, and ESEfinder prediction software predicted the variant located in the potential donor splice site. No other variants of interest were found in Glucagon (*GCG*) (preproglucagon) and related receptors, including Glucagon Receptor (*GCGR*) and Glucagon-Like Peptide 1 Receptor (*GLP1R*). In addition, no changes were observed in the copy number variant analysis.

FRG is characterized by urinary glucose excretion in the presence of normal blood glucose levels and the absence of renal tubular dysfunction. Defects in *SLC5A2* are the most frequent cause of FRG, with an estimated 90% of renal glucose reabsorption transported by SGLT2^37^. Familial renal glucosuria is commonly inherited as a dominant trait with incomplete penetrance^38,39^. In four families with either heterozygous or compound heterozygous *SLC5A2* variants, loss of transport capacity of up to 71.5% compared to wild-type controls and glucosuria quantified at 6–27 g/day were seen^39^. In one Finnish adult patient with suggested but not proven compound heterozygous *SLC5A2* variants, renal glucosuria was accompanied by postprandial hypoglycemia^37^.

Our findings of a rapid p-glucose half-life and severe glucosuria leading to occasional hypoglycemia suggest a functional significance of the *SGLT2* splice site variant identified. *SLC5A2* is predominantly expressed in the proximal tubules of the kidneys, but the functional mechanism leading to hypoglycemia in FGR may not exclusively be explained by urine glucose loss. *SLC5A2* is also expressed in pancreatic alpha cells, producing glucagon from proglucagon, but not in beta cells or the intestines^40^. In alpha cells, pharmacologic SGLT2 inhibition is reported to trigger glucagon secretion and hence gluconeogenesis^40^. We did not find increased fasting or glucose-stimulated p-glucagon but a hugely increased fasting p-GLP-1, which declined on OGTT.

GLP1 is mainly produced in the enteroendocrine L-cells in the distal ileum upon intestinal glucose stimulation via the SGLT1 transporter, encoded by *SLC5A1*. However, pancreatic alpha cells also produce GLP1 from proglucagon, especially at high glucose levels^41,42^. Our data suggest that the *SLC5A2* variant resulted in increased conversion of proglucagon to GLP1 but not to glucagon. Unfortunately, we did not obtain p-GLP1 during hypoglycemia to further elucidate the role of GLP1 in FRG. However, the increased p-GLP1 may, along with glucosuria, contribute to the fasting and postprandial hypoglycemia observed through complex interplay.

The mother was a healthy variant carrier. A broad phenotypic spectrum in FRG is well known^37^. It has been previously reported that not all individuals who carry the heterozygous variant presented with FRG, even among families with the exact variant, especially those who carried splicing variants^38^. Our data proposed an expansion of the genotype of FRG to include heterozygous *SLC5A2* variants with a phenotype in childhood of recurrent ketotic hypoglycemia, glucosuria and drastically increased fasting p-GLP1.

### The *Nek11* knockout model reduces glucose levels

A nonsyndromic, nonobese boy with a migraine was diagnosed with ketotic hypoglycemia at five years old (HH31, Table 1). Episodes with uneasiness and tremor, especially at the morning fast, had occurred for at least two years. His parents were healthy and nonconsanguineous, including his two elder brothers. His recurrent, severe attacks of migraine 2–3 times per month were unrelated to hypoglycemia at most occasions. Continuous glucose monitoring showed glucose values below 3.9 mmol/L almost every night.

On a 16-h fasting test, p-glucose fell to 2.3 mmol/L, blood ketones was 2.3 mmol/L, p-cortisol was 607 nmol/L, p-GH was 1.3 µg/L, and p-insulin was suppressed. He had normal liver enzymes but repeatedly mildly elevated p-ammonia of 61–94 (ref. 49) µmol/L and p-APTT of 44–46 (ref. 27–40) sec., decreased p-coagulation factors II + VII + X of 0.50–0.58 (ref. 0.70–1.30) U/L, intermittently decreased p-albumin of 32–41 (ref. 37–56) U/L and decreased p-protein of 58 (ref. 64–79) g/L. Hepatic ultrasound showed mild hepatopathy. Repeated hepatic fibro scans were high in the normal range for the adult reference interval, at 5.8 and 5.1 (ref. <6) kPa. A liver biopsy was not performed. By i.m. glucagon test, p-glucose did not increase but fell from 6.1 to 3.3 mmol/L at 90 minutes, where he was rescued for hypoglycemic symptoms. P-amino acids showed mild elevation of glutamine, ornithine, tyrosine, methionine, and phenylalanine. Taken together, biochemical liver effects with decreased glycogenolysis and mild changes in the hepatic ultrasound were observed.

Regular investigations included blood pressure, routine hematology, p-creatinine, urine metabolic screening, p-pyruvate, p-lactate, thyroid hormones, carnitine profile, ultrasound of the kidneys, muscle biopsy including electron microscopy, analysis of respiratory chain enzymes and pyruvate dehydrogenase and mitochondrial DNA deletions. His MRI of the brain with angiography was normal. A WISC-IV cognitive test showed variable performance with subscores from 66 to 93 (normal 100). The motor test movement ABC showed overall performance equaling one percentile. The boy had a continuous gross tremor of the hands.

Upon follow-up from 5–8 ½ years, height was normal (−1.5 SD), and bone age was delayed by 1.6 years. Repeat p-IGF1 values were 44–76 µg/L, equaling −1.3 SD. His p-IGFBP3 was repeatedly low, at 1133–1580 µg/L equaling approx. −2.3 SD. Peak p-GH was normal, at 15 µg/L by the arginine test and 27 µg/L by the clonidine stimulation test. At the age of 8 ½ years, an IGF-stimulation test (s.c. GH 30 µg/kg/d for four days, followed by 60 µg/kg/d for four days) failed to increase his p-IGFBP3 into the normal range; the peak was 2171 µg/L, and p-IGF1 increased to only 120 µg/L. There was the suspicion of hypoglycemia due to the liver involvement with the lack of response to i.m. glucagon test and the decreased production of IGFBP3, and hence, the expected low free p-IGF1 led to a trial of GH treatment in doses up to 60 µg/kg/d. The GH treatment succeeded in preventing him from reaching hypoglycemia.

Trio exome analysis identified a paternal heterozygous variant in the mitosis gene A-related kinase 11 gene (*NEK11*), and (p.Glu615Gly) was identified (HH31, Table 2). This variant replaced a highly conserved amino acid up to *Saccharomyces cerevisiae* and was predicted to be deleterious by SIFT, PolyPhen-2, and PROVEAN prediction software. In addition, no changes were observed in the copy number variant analysis, and no other candidate genes were identified. To date, there are no *NEK11* variants reported in humans.

NEK11 is a serine/threonine protein kinase reported to play a role in the S-phase checkpoint^43^. The knockout mouse phenotyping program (KOMP^2^) at the Jackson Laboratory was able to generate a *NEK11*^*tm1b(KOMP)Wtsi*^ mouse model lacking the critical exon 5 in *NEK11*^44^. These mice showed a significant reduction in glucose levels with no other phenotype reported (MGI:2442276; www.mousephenotype.org). In detail, 60% (n = 5) of female mice showed a level of 3.1–3.6 mmol/L compared to the wild-type level of 10.6 mmol/L, while 14% of male mice (n = 7) showed a level of 3.1 mmol/L compared to the wild-type level of 11.7 mmol/L. The variations in a hypoglycemia phenotype remained unexplained. Apart from the study of Noguchi *et al*., no studies on *NEK11* have been performed to the best of our knowledge. The tissue expression of *NEK11* is poorly described. *NEK11* expression in the liver would fit with the phenotype of the *NEK11*^*tm1b(KOMP)Wtsi*^ mice and with our proband, with the addition of a possible cerebral expression to explain the tremor and motor and cognitive deficits of our proband, which were more profound than suggested by the severity of his hypoglycemia.

The healthy father also carried the *NEK11* variant. As for mice, incomplete penetrance with the possibility of intrafamily variations may explain this finding.

Taken together, heterozygous *NEK11* variants may cause an incomplete penetrance phenotype with early childhood-onset cerebral affection with migraine, cognitive and motor deficits, and liver affection with ketotic hypoglycemia characterized by decreased glycogenolysis and decreased production of IGF1 and especially IGFBP3. The rescue of hypoglycemia by GH treatment suggested low free p-IGF1 to be causative of hypoglycemia. Our findings led to a referral to genetic counseling with expanded family investigations on the father’s side. Further studies are needed to understand the potential role of *NEK11* in hypoglycemia and cerebral function.

## Conclusion

In conclusion, using deep phenotyping and trio exome analyses, we identified DNA variants in four novel genes as the potential causes of IKH, suggesting that unexplained KH is a heterogeneous disorder to be split into at least four novel diseases. The identification of a possible or definite genetic cause may represent a large step forward in the management of unexplained KH in children in the context of personalized medicine. Our findings should encourage clinicians and researchers not to accept the term IKH before genetic investigations on GSD and other causes by exome sequencing. Finally, investigations into novel genes related to KH may lead to the identification of important novel drug targets and novel drug indications, including for the treatment of diabetes.

## Patients and Methods

### Patients

In a national base cohort, 36 patients up to 18 years old were recruited from the period 11/1/2015 to 10/30/2017, who presented with symptoms of hypoglycemia, at the Hans Christian Andersen Children’s Hospital, Denmark. Patients were evaluated based on Whipple’s triad criteria, including fasting and postprandial laboratory blood and urine tests for p-glucose and ketone bodies. Hyperketotic patients underwent an i.m. glucagon test, routine evaluation for growth hormone deficiency, adrenal insufficiency, and metabolic disorders. Some patients underwent muscle biopsy and other additional tests in search of a specific diagnosis. The inclusion criteria for the present study were patients with a diagnosis of ketotic hypoglycemia of unknown cause after the routine examination in the clinic. The exclusion criteria were nonketotic hypoglycemia and a genetic GSD diagnosis by clinical next-generation sequencing (NGS) panel analysis performed in patients with “idiopathic ketotic hypoglycemia”. Further exclusion criteria were the lack of informed consent for the present study.

### Ethics

The study was approved by the regional ethical committee of Southern Denmark (J.no. S-VF-20040235). All methods were performed in accordance with relevant guidelines and regulations. Informed consent was obtained from all parents and/or their legal guardians.

### Exome sequencing

DNA samples obtained from our patients and their family members were subjected to exome capture using Roche NimbleGen SeqCap EZ Exome 3.5 v Enrichment Kits (Roche, Hvidovre, Denmark) and sequenced on the Illumina HiSeq. 1500 platform. A read coverage is presented in detail in Table 3. Raw reads were processed using the Burrows-Wheeler Alignment tool (BWA-MEM) v. 0.7.12, and the GATK Best Practice pipeline v. 3.3-0 was used for variant calling.

**Table 3:** Target coverage and mean read depth.

Family ID	HH16	HH21	HH24	HH26	HH28	HH30	HH31	HH32	HH37
Target coverage (20x)(%)	92.1	56.3	89.1	92.1	92.7	89.7	87.2	85.9	92.5
Target coverage (10x )(%)	95.2	60.4	94	95.4	95.6	94.6	93.6	93.1	95.2
Mean read depth (x)	86	62	92	82	85	109	70	67	143

### Data analysis

**Figure 4:**
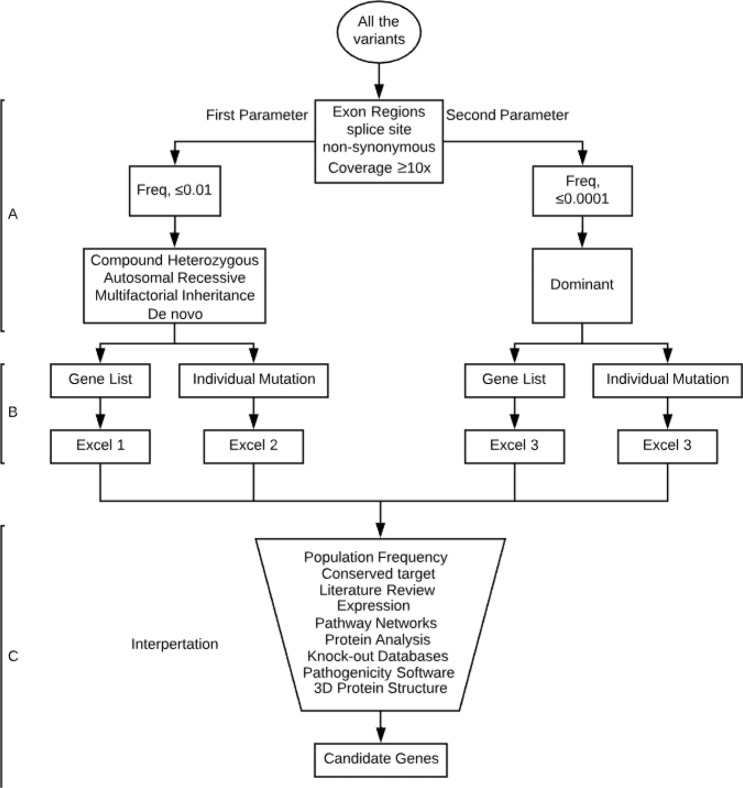
Exome data analysis strategy. Data filtering strategy; (**A**) Row data filter in all non-synonymous variants that presented within the exone regions or splicing sites including a minimum of 10X coverage. In parallel variant processed through two parameters; first parameter set with freq. of ≤0.01 for compound heterozygous, autosomal recessive, multifactorial inheritance, or de novo pattern while the second parameter set with freq. of ≤0.0001 for the dominant pattern. (**B**) Both parameters filtered against our gene list as well as individual mutations generated from step A will be kept without filtering. This step generates four excel files. (**C**) Data interpretation carried out manually for each file using multi-database with incorporated a considerable amount of judgment and extrapolation. This leads to generating a number of candidate genes that already known or newly discovered gene.

VarSeqTM (Golden Helix, Inc., Bozeman, MT) was used for downstream filtering. All variants were first filtered with a minimum of 10× coverage, nonsynonymous, and presented on the exome region or splice sites. The filtered variants were then processed twice, once for each parameter (Fig. 4- Step A); the first parameter, which covers the possibility of single nucleotide polymorphisms (SNPs), or small insertions and deletions (INDELs) in the form of a compound heterozygous, autosomal recessive, multifactorial inheritance, or de novo variants, set to a population frequency of ≤0.01 (gnomAD and ExAC)^45,46^. Notably, to not miss double de novo or a combination of one de novo and one inherited variant, all de novo variants ≤ 0.01 were included. The second parameter covers the dominant inheritance form of SNPs and INDELs and is set to a frequency of ≤0.0001 hypoglycemia disorders. Due to the enormous range of frequency presented for each cause of hypoglycemia disorders, a manual frequency filtering step for hypoglycemia cases and single de novo variants was applied during data interpretation taking into account the ethnicity and the clinical information.

In order to examine known causative genes that have been reported in the literature, including related genes and pathways, a gene list was generated consisting of 6264 genes that were categorized by disorders, pathways, expression, AmiGo terms, and other into 26 sublists. The entire list of 6264 gene can be found as Supplementary excel S1 online. This list was manually collected from different database sources covering all aspects of insulin- and glucose-related genes and disorders. This was done through an extensive literature review using PubMed, Ovid^®^, GeneCards^®^, and National Center for Biotechnology Information (NCBI). Gene and protein expression databases such as BioGPS and The Human Protein Atlas were used. Protein interactions and gene network databases such as AmiGO, BioGRID, GIANT, KEGG, and Reactome were also used. Knockout mouse databases such as MGI and IMPC were also used. The entire databases used can be found as Supplementary Table S2 online. However, filtering against the gene list will not replace the manual screening for all variants called; therefore, we did not consider the results of our gene list alone. Once the raw data were obtained, they were filtered and investigated individually. As shown in Fig. 4, variants went through serial steps ending with a single nucleotide polymorphism variant as a potential explanation. Pathogenicity scores were determined by SIFT, PolyPhen-2, PROVEAN, NetGene2, MaxEnt, and ESEfinder.

### Protein sequence analysis

The three-dimensional protein structure of candidate genes was analyzed using PyMOL v.1.7.4 (Schrödinger, New York, USA). (*IGF2BP1*-PDB-ID: 3KRM).

### Sanger sequencing

Variants detected with less than 50x coverage or presented in *de novo* form were validated with Sanger using the BigDye Terminator v. 3.1 cycle sequencing kit and an ABI 3730xl capillary sequencer (Thermo Fischer, Naerum, Denmark). All variants of interest had a coverage of above 50x by exome sequencing including *NCOR1*, *SLC5A2*, and *NEK11* with 64×, 78×, and 96×, respectively. The *de novo IGF2BP1* variant was the only one fits our criteria for validation. The *IGF2BP1* primer was purchased from pxlence.com (catalog no.: PXL-A0116969).

## Supplementary information

Supplementary Information.

Supplementary Dataset 1.

## Data Availability

All data generated are available upon request. One Excel file contains a list of genes and its sublists used during filtering (File name: Analysis Gene List.xlsx).
