# Supplementary material for: Exome sequencing revealed DNA variants in NCOR1, IGF2BP1, SGLT2 and NEK11 as potential novel causes of ketotic hypoglycemia in children
Source: Sci Rep. 2020 Feb 7;10:2114. doi: 10.1038/s41598-020-58845-3 (PMC7005888; doi:10.1038/s41598-020-58845-3)
Supplement: Supplementary file 1 — Supplementary Information. [file 41598_2020_58845_MOESM1_ESM.docx]

**Exome sequencing revealed DNA variants in *NCOR1*, *IGF2BP1*, *SGLT2* and *NEK11* as potential novel causes of ketotic hypoglycemia in children**

**Authors:** Yazeid Alhaidan ^1,2,3,4 *^, Martin J. Larsen ^1,2^, Anders Jørgen Schou ^5^, Maria H. Stenlid ^6^, Mohammed A. Al Balwi ^3,4^, Henrik Thybo Christesen ^2,5,7^**^†^**, Klaus Brusgaard ^1,2,8^**^†^**.

**Affiliations:** ^1^ Department of Clinical Genetics, Odense University Hospital, 5000 Odense C, Denmark. ^2^ Department of Clinical Research, Faculty of Health Sciences, University of Southern Denmark, 5000 Odense C, Denmark. ^3^ Department of Medical Genomics Research, King Abdullah International Medical Research Center, Riyadh, 11426, Saudi Arabia. ^4^ King Saud bin Abdulaziz University for Health Sciences, Riyadh, Saudi Arabia. ^5^ Hans Christian Andersen Children’s Hospital, Odense University Hospital, 5000 Odense C, Denmark. ^6^ Department of Paediatric Endocrinology, Uppsala University Children's Hospital, Uppsala, Sweden. ^7^ Odense Pancreases Center, [www.OPAC.nu](http://www.OPAC.nu). ^8^ Near East University, Nicosia, Cyprus.

***Corresponding author:** Yazeid Alhaidan, Department of Clinical Genetics, Odense University Hospital, J.B. Windsløws Vej 4, DK-5000 Odense, Denmark. Tel.: +45 65412822 Fax: +45 65414875 e-mail: [yazeid@mail.net.sa](mailto:yazeid@mail.net.sa)

**^†^Footnotes:** Henrik Thybo Christesen and Klaus Brusgaard contributed equally to this work.

| Table S1. Number of genes collected for each category in our gene list. | |
| --- | --- |
| Groups | Number of genes |
| Insulin-glucose related genes | 801 |
| Diabetic and related genes | 171 |
| Hyperinsulinemic and related genes | 136 |
| Insulinoma and related genes | 224 |
| Hypoglycemia and related genes | 556 |
| Glycolysis – Gluconeogenesis and related genes | 596 |
| Glycogen storage disease and related genes | 34 |
| Congenital disorders of glycosylation and related genes | 111 |
| Insulin secretion and pathways and related genes | 295 |
| Mitochondria and related genes (mtDNA and nDNA) | 1054 |
| Glucose and related genes | 2501 |
| Glucagon and related genes | 182 |
| Glycogen and related genes | 344 |
| Calcium and related genes | 179 |
| Fatty Acid and related genes | 243 |
| Up-regulating insulin genes | 101 |
| Down-regulating insulin genes | 65 |
| Pancreatic secretion genes | 96 |
| ABCC8 regional genes | 76 |
| PI3K-Akt signaling pathway genes | 339 |
| Adipocytokine signaling pathway genes | 78 |
| Digesting & Absorbing genes | 174 |
| Highly pancreatic expression genes | 94 |
| Highly liver expression genes | 434 |
| Knock out genes causing abnormality in glucose level | 244 |
| Knock out genes causing abnormality in insulin level | 122 |
| Total (after deleting overlap genes) | **6264** |

**Excel file contains all the genes names used during filtering can be downloaded online (File name: Analysis Gene List.xlsx).**

|  | Databases | Web link |
| --- | --- | --- |
| literature review | National Center for Biotechnology Information (NCBI) including Pubmed | https://www.ncbi.nlm.nih.gov/  https://www.ncbi.nlm.nih.gov/pubmed/ |
|  | Ovid® | https://www.ovid.com/ |
|  | GeneCards® | https://www.genecards.org/ |
| Gene and protein expression | BioGPS | http://biogps.org/ |
|  | The Human Protein Atlas | https://www.proteinatlas.org/ |
| Protein/gen interactions and networks | AmiGO | http://amigo.geneontology.org/ |
|  | BioGRID | https://thebiogrid.org/ |
|  | GIANT | http://giant.princeton.edu/ |
|  | KEGG | https://www.kegg.jp/ |
|  | Reactome | https://reactome.org/ |
| Knock-out mice | MGI | http://www.informatics.jax.org/ |
|  | IMPC | http://www.mousephenotype.org/ |
| Protein information | UniProt | https://www.uniprot.org/ |
| Mitochondrial | Human Mitochondrial Genome | http://www.mtdb.igp.uu.se/ |
|  | MITOMAP | https://www.mitomap.org/MITOMAP |
| **Table S2; Database and web sources for gene list collection and data interpretation.** | | |
